# Verdi opera training intensity and vocal expressiveness: the sequential mediating roles of role empathy and dramatic tension in a cross-sectional study

**DOI:** 10.3389/fpsyg.2026.1866865

**Published:** 2026-09-28

**Authors:** Xia Li, Tong Zhu

**Affiliations:** 1 Keimyung College University (KMCU), Daegu, Republic of Korea; 2 Keimyung University (KMU), Daegu, Republic of Korea

**Keywords:** chain mediation model, dramatic tension, role empathy, Verdi opera training, vocal expressiveness

## Abstract

**Inroduction:**

Vocal expressiveness is a key component of opera performance, yet the psychological mechanisms through which Verdi opera performance training enhances vocal expressiveness remain insufficiently understood. This study examined the relationship between Verdi opera performance training and vocal expressiveness, with particular attention to the roles of role empathy and perceived dramatic tension.

**Methods:**

A total of 558 vocal music students who had received systematic training in Verdi opera participated in the study. Questionnaire data were collected and analysed using bootstrap-based mediation analysis to test the proposed chain mediation model.

**Results:**

The findings indicated that Verdi opera performance training was positively associated with vocal expressiveness. Both role empathy and perceived dramatic tension were significantly associated with this relationship. In addition, the proposed sequential indirect association through role empathy and perceived dramatic tension was supported.

**Discussion:**

These findings provide empirical evidence for the psychological mechanisms associated with vocal expressiveness and suggest that integrating technical training with role understanding and dramatic interpretation may enhance opera education. The study contributes to the understanding of vocal artistry from a psychological perspective and offers practical implications for opera training in higher music education.

## Introduction

Among the canon of Western opera, the works of Giuseppe Verdi are widely recognized for their profound humanism, intense emotional tension, and nuanced portrayal of human complexity. Verdi’s music is not merely a combination of melody and harmony, but a form of “drama in sound” (Marvin, 2002), carrying substantial dramatic and psychological depth. For vocal students, mastering the technical demands of Verdi’s operas is only the initial stage; the greater challenge lies in using the voice to convey the inner life of the character, achieving a close integration of technical proficiency and artistic expression—namely, vocal expressiveness (Fountoukidou et al., 2022; Marquezin et al., 2015). Vocal expressiveness is not simply the accumulation of isolated techniques, but a holistic form of artistic expression in which emotional experience, dramatic understanding, and vocal control are closely integrated (Huron, 2016; Thompson et al., 2023; Juslin, 2025). However, the systematic development of this advanced ability—particularly in works characterized by intense dramatic conflict and layered emotions—remains a key issue in vocal education that requires further investigation.

Traditional vocal pedagogy typically emphasizes foundational training in technique, intonation, and rhythm; although this is generally regarded as providing the necessary basis for expressiveness, it often overlooks deeper exploration of a character’s psychological dimensions and the immersive nature of the dramatic context (Helding, 2007; Reid, 2002). In recent years, researchers have increasingly recognized the importance of empathy in artistic and musical engagement (Clarke et al., 2015). For performers, empathy takes on a particular significance because they are required to engage with the perspectives and experiences of the characters they portray. Gallagher and Gallagher (2020) in their discussion of actors’ empathy for their characters, describe this relationship as one involving imagination and an understanding of the character from within the performance. In the present study, role empathy therefore refers to the singer’s capacity to understand, experience, and internalize a character’s emotions, motivations, and perspective. Research in music also suggests that empathy is relevant to musical engagement and performance. Clarke et al. (2015) describe empathy as an important dimension of musical experience and interpersonal understanding, while Babiloni et al. (2012) found an association between empathy traits and neural activity related to emotional empathy in professional musicians. Research conducted in a Chinese context has likewise demonstrated a relationship between interpersonal empathy and music empathizing (Zhao et al., 2022). Together, these findings suggest that role-specific empathic engagement may be relevant to how performers understand and communicate emotional content through music.

This issue is particularly relevant to Verdi’s operas, where vocal performance is closely connected to the dramatic circumstances and psychological states of the characters. Characters are often situated within complex historical and ethical conflicts, and singers are required to communicate these conflicts through both musical interpretation and characterization. If singers focus solely on accurate vocal production without embodying the character, their performance may be less likely to be associated with a strong expressive impact. Role empathy may therefore provide one means through which engagement with Verdi opera training is associated with vocal expressiveness: a deeper understanding of a character’s emotional situation and motivations may be associated with a greater ability to convey these qualities through vocal performance.

However, role empathy alone does not fully account for vocal expressiveness. Another defining feature of Verdi’s operas is their strong dramatic tension (Freiman, 2025; Piazzoli, 2017). This tension is associated with the interaction between musical structure, the libretto, and may be reflected in a sustained dramatic progression (Wang, 2024; Markantonis, 2023). Singers must not only understand their roles but also actively construct and convey this tension. This suggests that emotional experience associated with role empathy may also be related to structured dramatic expression.

Existing research suggests that the ability to perceive and construct dramatic tension distinguishes professional opera singers from students (Bourne and Kenny, 2016; Sandgren, 2005). From this perspective, role empathy and perceived dramatic tension may represent related but distinct aspects of expressive performance. Role empathy concerns the singer’s engagement with the character’s emotional and psychological world, whereas perceived dramatic tension concerns the singer’s awareness of the developing dramatic forces within the performance. A stronger empathic engagement with the role may thus be accompanied by a heightened perception of dramatic tension, which may subsequently be associated with greater vocal expressiveness.

Based on this rationale, this study proposes a chain mediation model to examine the psychological pathways linking the intensity of Verdi opera performance training with vocal expressiveness. The model suggests that specific Verdi opera training practices (e.g., textual analysis, situational rehearsal, and staged performance practice) may be associated with higher levels of role empathy, which may in turn be associated with a greater ability to perceive and construct dramatic tension. These associations form a proposed sequential indirect pathway of “role empathy → dramatic tension” that may help explain variation in vocal expressiveness. The proposed model therefore focuses on how psychological engagement with the role and the dramatic situation may help explain the relationship between opera training and expressive performance.

This study empirically tests the proposed chain mediation model. The sample consists of vocal music students who have received systematic Verdi opera training. Role empathy, perceived dramatic tension, and vocal expressiveness were quantitatively measured, and the bootstrap method was used to examine the mediation effects.

The following hypotheses are proposed:

*H1*: Verdi opera performance training intensity is positively associated with vocal expressiveness.

*H2*: Verdi opera performance training intensity is positively associated with role empathy.

*H3*: Verdi opera performance training intensity is positively associated with perceived dramatic tension.

*H4*: Role empathy is positively associated with perceived dramatic tension.

*H5*: Role empathy and perceived dramatic tension sequentially mediate the relationship between Verdi opera performance training intensity and vocal expressiveness.

This study may provide a theoretical basis for understanding the development of vocal expressiveness and may offer practical implications for optimizing opera training in higher music education (Figure 1).

**Figure 1:**
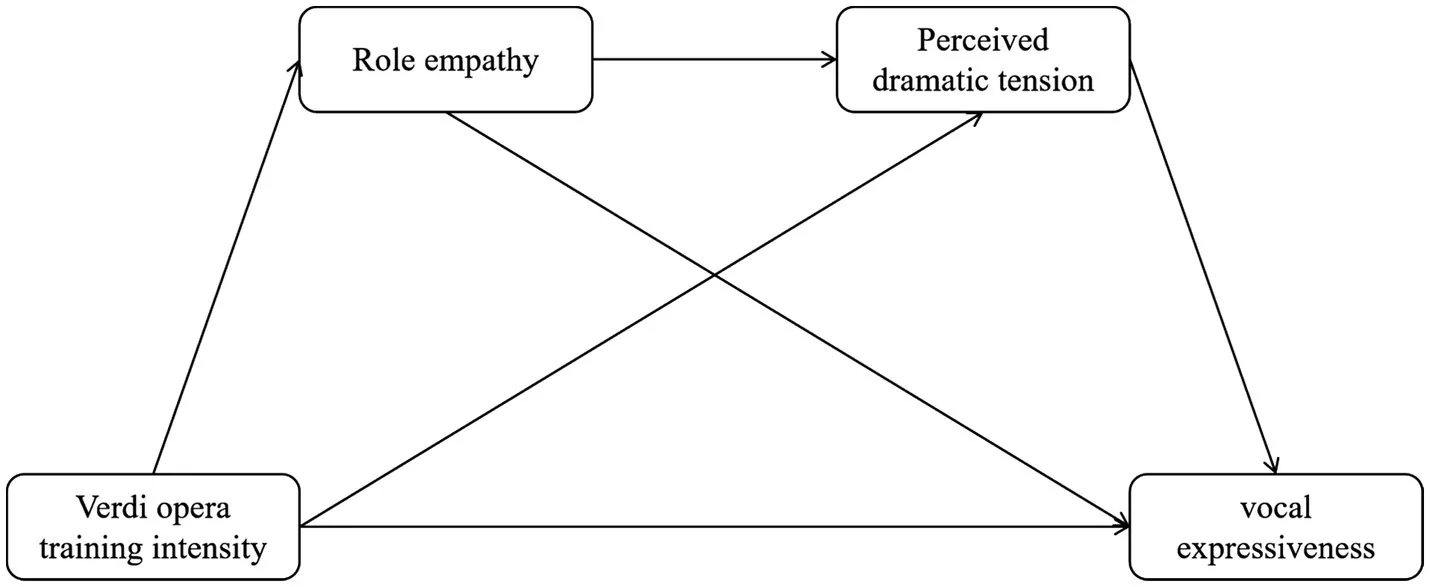
Proposed chain mediation model linking Verdi opera training intensity, role empathy, perceived dramatic tension, and vocal expressiveness.

## Materials and methods

### Participants

This study used a combination of convenience and purposive sampling to recruit participants from vocal and opera departments at three music conservatories in China. The inclusion criteria were: (1) full-time undergraduate or postgraduate students majoring in vocal performance (bel canto); (2) completion of at least one semester of a course focused on Verdi operas; and (3) voluntary participation with informed consent provided.

Participants were recruited from vocal and opera departments at three music conservatories in China, here anonymized as Conservatories A, B, and C. Eligible students were identified through departmental class rosters and course enrolment lists, and all students who met the inclusion criteria were invited to participate. Across the three institutions, 600 eligible students were identified and invited, 578 questionnaires were returned, and 558 valid responses were retained after screening.

Data screening excluded invalid responses with more than 10% missing data (*n* = 12), excessively short response times (< 300 s; *n* = 0), or obvious repetitive response patterns (*n* = 8), resulting in 558 valid responses (93.0%).

The sample included 281 males (50.4%) and 277 females (49.6%), aged 18–26 years (M = 21.95, SD = 2.54). Among them, 259 were undergraduates (46.4%) and 299 were postgraduate students (53.6%). All participants reported at least five hours per week of Verdi opera-related training. The study was approved by the Ethics Committee of Keimyung University (Approval No.: 2026-12-31-J-E-022035), and all procedures followed the Declaration of Helsinki.

### Procedure

Questionnaires were distributed during the spring semester of 2025 in courses focused on Verdi opera performance and interpretation at the participating conservatories. After a brief introduction by course instructors, students accessed the survey through an online questionnaire platform and completed it individually after reading the study information and providing informed consent. All measures were presented in a fixed order. Before formal distribution, the questionnaire was piloted with a small group of vocal students to ensure item clarity and an appropriate completion time. To reduce careless responding, duplicate submissions were restricted and all records were screened for missingness, extremely short completion time, and obvious repetitive response patterns during data cleaning.

#### Verdi opera performance training intensity (VOPTI)

This scale was developed by the research team based on a literature review and expert interviews, with the aim of assessing the intensity of participants’ systematic training in Verdi opera over a specific period (the past academic term). In this study, Verdi opera performance training was defined as structured learning activities related to Verdi’s operatic works, including vocal technique practice, aria interpretation, repertoire study, character analysis, and dramatic performance preparation conducted through formal music education and performance courses.

The Verdi opera repertoire involved in participants’ training included representative works such as *La traviata*, *Rigoletto*, *Aida*, *Il trovatore*, and *Otello*. Through Verdi opera-related courses, including *Opera Performance*, *Italian Opera Repertoire*, *Western Opera Interpretation*, and *Opera Character Analysis*, participants engaged in systematic learning of Verdi’s vocal style, musical structures, dramatic characters, and stage interpretation. These training activities provided the contextual foundation for developing role empathy and perceiving dramatic tension during opera performance.

The scale comprises five items, such as “How many hours per week do I devote specifically to practising Verdi opera arias?” and “How often do I engage in in-depth analysis of Verdi opera roles (e.g. background, motivation, psychology)?” The scale employs a mixed scoring method, with some items using a 5-point Likert scale (1 = never, 5 = always) and others requiring the recording of specific durations. The total scale score is calculated using a weighted average; a higher score indicates greater training intensity.

To strengthen the content basis of the instrument, the item pool was developed with reference to prior work on structured music practice, rehearsal engagement, and performance preparation. In this study, the Cronbach’s α coefficient for this scale was 0.799.

#### Role empathy

Role empathy was measured using the “Perspective Taking” and “Empathic Concern” subscales of the Interpersonal Reactivity Index (IRI; Davis, 1980). This instrument has been widely used and discussed in music and performing-arts research (e.g., Clarke et al., 2015; Babiloni et al., 2012; Zhao et al., 2022). The scale includes 14 items rated on a 5-point Likert scale (0–4). Scores were summed, with higher values indicating greater role empathy. Example items included statements comparable to “I try to look at everybody’s side of a disagreement before I make a decision” and “I often have tender, concerned feelings for people less fortunate than me”, adapted here to reflect performers’ role-related empathic engagement. Cronbach’s α was 0.901.

#### Perceived dramatic tension

Perceived Dramatic Tension (PDT) was measured using a scale developed for this study, based on prior research on musical performance, theatricality, and dramatic structure (e.g., Piazzoli, 2017; Markantonis, 2023; Freiman, 2025). The scale includes seven items assessing the perception of dramatic conflict and emotional dynamics in Verdi operas. Example items asked whether the singer could clearly perceive dramatic conflict across scenes and whether changes in musical and emotional momentum were strongly felt during performance preparation. Responses were rated on a 7-point Likert scale (1–7), and scores were averaged, with higher values indicating stronger perceived dramatic tension. Cronbach’s α was 0.794.

#### Vocal expressiveness

Vocal expressiveness was assessed using a self-report scale developed based on Huron’s (2016) multidimensional framework. The scale includes 12 items assessing emotional authenticity, timbral variation, phrasing coherence, and overall performance impact. Example items assessed whether the singer could convey authentic emotion, shape phrases coherently, vary vocal colour according to the dramatic situation, and generate an overall sense of expressive impact in performance. Responses were rated on a 6-point Likert scale (1–6), and scores were averaged, with higher values indicating greater expressiveness. Cronbach’s α was 0.894.

#### Statistical analysis

Data analysis was conducted using SPSS 26.0 and the PROCESS macro (Model 6). Descriptive statistics and Pearson correlation analyses were first performed to examine relationships among the main variables. The proposed chain mediation model (Verdi opera performance training → role empathy → perceived dramatic tension → vocal expressiveness) was then tested using the bootstrap method (Hayes, 2017).

Regression analyses were conducted sequentially to estimate direct and indirect effects. A bias-corrected bootstrap procedure with 5,000 resamples was used to calculate 95% confidence intervals (CI) for indirect effects. Mediation was considered significant when the CI did not include zero. Variance inflation factor (VIF) values were additionally examined to assess potential multicollinearity among predictors in the regression models. All tests were two-tailed.

## Results

Descriptive statistics and Pearson correlation analyses were conducted for all variables, as presented in Table 1. The absolute values of skewness and kurtosis were below 2 for all variables, indicating approximate normality and suitability for parametric analyses.

**Table 1:** Descriptive statistics and correlations among variables (*N* = 558).

Variable	M	SD	1	2	3	4
1. Training intensity	2.14	0.97	1			
2. Role empathy	20.91	13.90	0.826	1		
3. Dramatic tension	2.80	1.18	0.778	0.792	1	
4. Vocal expressiveness	2.53	1.07	0.829	0.842	0.803	1

All correlation coefficients were significant at *p* < 0.001. Absolute skewness and kurtosis values were below 2 for all focal variables.

Correlation analysis showed significant positive associations among the variables. Verdi opera performance training intensity was positively correlated with vocal expressiveness (r = 0.829, *p* < 0.001), supporting H1. It was also positively correlated with role empathy (r = 0.826, *p* < 0.001) and perceived dramatic tension (r = 0.778, *p* < 0.001), supporting H2 and H3. Role empathy was positively correlated with perceived dramatic tension (r = 0.792, *p* < 0.001), supporting H4. In addition, both role empathy (r = 0.842, *p* < 0.001) and perceived dramatic tension (r = 0.803, *p* < 0.001) were positively correlated with vocal expressiveness. Overall, all variables were significantly positively correlated.

Multicollinearity diagnostics further showed acceptable levels of shared variance among predictors. Specifically, VIF values ranged from 1.00 to 3.86 across the regression models, remaining below commonly used cut-off values and suggesting that multicollinearity was not a serious concern.

### Testing for chain mediation

The results of the regression analyses are presented in Table 2. Verdi opera performance training was positively associated with role empathy (*β* = 0.826, *p* < 0.001). In the model predicting perceived dramatic tension, both Verdi opera performance training (*β* = 0.388, *p* < 0.001) and role empathy (*β* = 0.473, *p* < 0.001) were positively associated with perceived dramatic tension.

**Table 2:** Regression results for the chain mediation model (*N* = 558).

Dependent variable	Predictor	β	t	*p*	R^2^	ΔR^2^
Role empathy (M1)	Verdi opera training (X)	0.826	34.531	< 0.001	0.685	0.685
	Gender	0.036	1.495	0.135		
	Age	0.037	1.543	0.123		
	Educational level	−0.021	−0.868	0.386		
Dramatic tension (M2)	Verdi opera training (X)	0.388	8.987	< 0.001	0.676	0.348
	Role empathy (M1)	0.473	10.963	< 0.001		
	Gender	−0.006	−0.253	0.800		
	Age	−0.024	−0.969	0.333		
	Educational level	0.010	0.417	0.677		
Vocal expressiveness (Y)	Verdi opera training (X)	0.319	8.523	< 0.001	0.787	0.111
	Role empathy (M1)	0.374	9.676	< 0.001		
	Dramatic tension (M2)	0.259	7.497	< 0.001		
	Gender	0.004	0.207	0.836		
	Age	0.003	0.169	0.866		
	Educational level	0.007	0.353	0.724		

Standardized coefficients (β) are reported. VIF values ranged from 1.00 to 3.86 across the three regression models.

In the final model predicting vocal expressiveness, Verdi opera performance training (*β* = 0.319, *p* < 0.001), role empathy (*β* = 0.374, *p* < 0.001), and perceived dramatic tension (*β* = 0.259, *p* < 0.001) were all positively associated with vocal expressiveness. The direct effect of training was smaller than the total effect (*β* = 0.830), suggesting the presence of a statistically significant indirect association.

Bootstrap analyses (5,000 resamples) were conducted to estimate indirect effects and their 95% confidence intervals (CI). As shown in Table 3, the total effect of Verdi opera performance training on vocal expressiveness was 0.830 (*p* < 0.001), and the direct effect remained significant (*β* = 0.319, 95% CI [0.246, 0.393]).

**Table 3:** Bootstrap results for indirect effects in the chain mediation model (N = 558).

Effect	Path	Effect size	SE	LLCI	ULCI	% of total effect
Total effect	X → Y	0.830	0.024	0.783	0.876	100.0%
Direct effect	X → Y	0.319	0.038	0.246	0.393	38.5%
Indirect effect 1	X → M1 → Y	0.309	0.040	0.232	0.389	37.2%
Indirect effect 2	X → M2 → Y	0.100	0.018	0.066	0.138	12.1%
Indirect effect 3	X → M1 → M2 → Y	0.101	0.018	0.069	0.140	12.2%
Total indirect effect	—	0.510	0.038	0.438	0.586	61.5%

X = Verdi opera performance training; M1 = role empathy; M2 = perceived dramatic tension; Y = vocal expressiveness. LLCI = lower limit of the 95% confidence interval; ULCI = upper limit of the 95% confidence interval.

All indirect effects were statistically significant. The indirect effect via role empathy (X → M1 → Y) was 0.309 (95% CI [0.232, 0.389]), accounting for 37.2% of the total effect. The indirect effect via perceived dramatic tension (X → M2 → Y) was 0.100 (95% CI [0.066, 0.138]), accounting for 12.1% of the total effect. The sequential indirect effect (X → M1 → M2 → Y) was 0.101 (95% CI [0.069, 0.140]), accounting for 12.2% of the total effect.

Pairwise comparisons of indirect effects showed that the indirect effect via role empathy (Ind1) was significantly larger than both the indirect effect via perceived dramatic tension (Ind2) and the sequential indirect effect (Ind3) (C1: 95% CI [0.114, 0.310]; C2: 95% CI [0.110, 0.303]). No significant difference was found between Ind2 and Ind3 (C3: 95% CI [−0.047, 0.041]). Overall, these findings are consistent with the proposed chain mediation model.

Bootstrap confidence intervals are based on 5,000 resamples. Effects are considered significant when the 95% confidence interval does not include zero.

## Discussion

This study examined a chain mediation model to explain how Verdi opera performance training relates to vocal expressiveness. The results show that training was directly and indirectly associated with vocal expressiveness, with role empathy and perceived dramatic tension forming a significant sequential indirect association. These findings provide empirical evidence regarding the psychological factors associated with vocal expressiveness and offer practical implications for vocal pedagogy.

The findings highlight the role of role empathy as an important link between training and vocal expressiveness. Verdi opera performance training was positively associated with role empathy, suggesting that structured activities such as character analysis and situational rehearsal may be associated with performers’ understanding of their roles. This is consistent with neuroaesthetic perspectives on empathy, which propose that engagement with characters can activate shared emotional representations (Molnar-Szakacs and Overy, 2006; Toader et al., 2023).

In this study, role empathy showed a substantial indirect association with vocal expressiveness, accounting for a large proportion of the total effect. This finding is consistent with previous research emphasizing emotional authenticity as a key component of expressive performance (Fountoukidou et al., 2022; Marquezin et al., 2015). It also is consistent with the pedagogical principle of integrating emotional understanding with vocal production.

An additional contribution of this study is the identification of perceived dramatic tension as part of the indirect association between role empathy and vocal expressiveness. The results indicate that role empathy was positively associated with perceived dramatic tension, suggesting that a deeper understanding of the character may be associated with greater awareness and construction of dramatic dynamics within performance.

Furthermore, perceived dramatic tension independently showed a significant indirect association in the relationship between training and vocal expressiveness. This implies that emotional understanding alone may not be sufficient to account for vocal expressiveness; it may also be associated with structured expressive output. This finding is consistent with previous studies highlighting the role of dramatic tension in shaping performance quality (Piazzoli, 2017; Freiman, 2025; Bourne and Kenny, 2016).

The results further are consistent with a sequential indirect pathway from Verdi opera performance training to vocal expressiveness through role empathy and perceived dramatic tension.

In this context, training may be associated with greater performers’ engagement with the character and may also be associated with the construction of dramatic tension, which together may be related to expressive performance. This extends previous research that has typically examined single mediating factors in expressive performance and opera-related training contexts.

The findings have practical implications for vocal training in higher music education. Traditional approaches may emphasize technical skills while giving less attention to the integration of dramatic and emotional elements (Helding, 2007; Reid, 2002). The present results suggest that effective teaching should incorporate both dimensions.

Specifically, instructors may consider combining technical training with activities that support role understanding, such as textual analysis and contextual interpretation. In addition, guidance on how to translate internal emotional experience into external performance features—such as dynamics, phrasing, and vocal colour—may be associated with improved expressive outcomes.

This study has several limitations. First, the cross-sectional design limits causal inference. Future research could adopt longitudinal or experimental designs to better establish causal relationships. Second, as all measures were based on self-report, common method bias may be present. Future studies could incorporate multi-source data, such as expert ratings or physiological measures. The revised VIF diagnostics suggest that multicollinearity was not a serious concern in the present models. Third, the sample consisted primarily of students from conservatories in China, which may limit generalisability. Future research should examine more diverse cultural and educational contexts. Finally, additional mediating or moderating variables, such as self-efficacy or performance anxiety, could be explored.

## Conclusion

This study examined a sequential mediation model to explain how Verdi opera training relates to vocal expressiveness. The findings indicate that training was directly and indirectly associated with vocal expressiveness, with significant indirect associations observed through role empathy and perceived dramatic tension.

Training was positively associated with role empathy, which was in turn positively associated with perceived dramatic tension, and both variables were positively associated with vocal expressiveness. These results suggest a pattern of sequential associations among training, psychological factors, and expressive outcomes.

This study provides quantitative support for pedagogical approaches that integrate emotional understanding with vocal training. The findings suggest that opera training may benefit from greater integration of technical and expressive components.

## Data Availability

The raw data supporting the conclusions of this article will be made available by the authors, without undue reservation.
